# Coupling among Electroencephalogram Gamma Signals on a Short Time Scale

**DOI:** 10.1155/2010/946089

**Published:** 2010-07-29

**Authors:** Michael P. McAssey, Fushing Hsieh, Anne C. Smith

**Affiliations:** ^1^Department of Statistics, University of California Davis, MSB 4118 One Shields Avenue, Davis, CA 95616, USA; ^2^Department of Anesthesiology and Pain Medicine, University of California TB-170, One Shields Avenue, Davis, CA 95616, USA

## Abstract

An important goal in neuroscience is to identify instances when EEG signals are coupled. We employ a method to measure the coupling strength between gamma signals (40–100 Hz) on a short time scale as the maximum cross-correlation over a range of time lags within a sliding variable-width window. Instances of coupling states among several signals are also identified, using a mixed multivariate beta distribution to model coupling strength across multiple gamma signals with reference to a common base signal. We first apply our variable-window method to simulated signals and compare its performance to a fixed-window approach. We then focus on gamma signals recorded in two regions of the rat hippocampus. Our results indicate that this may be a useful method for mapping coupling patterns among signals in EEG datasets.

## 1. Introduction

 Current neuroscience research is focused not only on identification of brain regions associated with particular cognitive tasks, but also on how those regions interact during the execution of the these tasks on a short time scale [1–3]. In this paper, we investigate methods for identifying brief instances in time when groups of gamma-band signals (40–100 Hz) extracted from electroencephalogram (EEG) recordings become synchronized. Our methods are developed to address the specific problem of analyzing EEG recordings from the rat hippocampal formation. Recent studies [2] have demonstrated dynamic coordination at these frequencies between the dentate gyrus, CA1, and CA3 during tasks with high cognitive demand and during REM sleep.

Typically in situations where a signal is suspected to be changing with time, methods from signal analysis, such as the short-time Fourier transform, can be applied quite successfully. For pairs of signals, *coherency*, a measure of signal coherence in the frequency domain, can be computed with confidence bounds, using for example a multitaper [4]. In both of these cases software has been developed to compute these quantities. For example, the spectrogram function in Matlab [5] computes the short-time Fourier transform along segments of an individual signal, and the coherence function in the Chronux package [6] computes the windowed coherence between pairs of signals. However, for signals with synchrony lasting just a few cycles at a time, frequency-domain methods can be less sensitive and may yield unrealistically large confidence bounds. Alternative methods for detecting synchronization of neuroelectric signals are based on transient phase locking [7, 8]. For the case of many EEG signals, other approaches include Granger causality [9], autoregressive modeling [10], and Bayesian networks [11]. However, these may be limited in their ability to detect only brief instances of synchrony.

One of the challenges in analysis of biological rhythms is that the signal frequency can be quite variable. We develop here a method that computes synchrony among multiple signals on the time scale of milliseconds and which yields, not just pairwise computations, but a joint result for all signals. We do this by making use of a small time-varying sliding window to compute pairwise cross-correlations, and then by using the Expectation-Maximization (EM) algorithm [12] applied to a mixed multivariate beta model to identify groups of gamma signals that are highly synchronized at any instant.

Our technique consists of two steps: first, we select one of the available gamma signals as the basis for providing the sliding measurement window and the temporal axis for computation of the instantaneous coupling between that dimension and each of the other dimensions and second, we use the EM algorithm to classify the collection of instantaneous coupling measurement vectors into a fixed number of states representing different occasions of gamma-band binding among brain regions.

The effectiveness and reliability of our methods are tested on simulated data. Our technique is then applied to a nine-channel EEG data set recorded from tetrodes implanted in the Medial Entorhinal Cortex (MEC) and the CA1 cell layer of the hippocampus in a rat's brain.

## 2. Methods

We assume that subsets of gamma signals extracted from EEG recordings using a band-pass filter are subject to instants of synchrony on the order of a few cycles, after which they become unsynchronized. We call this phenomenon *instantaneous coupling* (IC) and develop a method for quantifying it.

### 2.1. Computation of Instantaneous Coupling between Two EEG Signals

Since we are looking for synchrony on very short time scales and since biological signals are prone to variability, we focus here on finding an appropriate time scale for computing coupling between pairs of signals that adjusts over time based on the changing frequency of the signals.

We would like to compute a sequence of IC estimates between two bandpassed oscillating time signals in the gamma range (40–100 Hz), say *X* = {*x*_*t*_}_*t*=1_^*T*^ and *Y* = {*y*_*t*_}_*t*=1_^*T*^, throughout a given epoch consisting of *T* time points. Our approach is similar to the procedure given in [13], but we use a time-varying window. The amplitude, frequency, and phase of each signal vary from one instant to the next, not necessarily independently. We choose one of the signals, say *X*, as the base signal. We then partition the entire epoch based on the *N* ≪ *T* zero crossings of *X*, which we denote by *Z*_1_, *Z*_2_,…, *Z*_*N*_. We regard each interval [*Z*_*i*_, *Z*_*i*+2_], *i* = 1,2,…, *N* − 2, as a full cycle, and thus [*Z*_*i*_, *Z*_*i*+1_] is a half-cycle, *i* = 1,2,…, *N* − 1. The duration of a cycle may thus vary significantly throughout the epoch.

Let the integer *w* indicate the window size, that is, the number of half cycles of the base signal to be used in determining the duration of an “instant” when estimating the instantaneous coupling between *X* and *Y*. Choosing a small value for *w* not only enables us to discuss the dynamics of signal coupling among brain regions on a very short time scale, but also provides us with approximate stationarity in the signals. However, if *w* is chosen too small, the IC may be overestimated due to the sparsity of information. If *w* is chosen too large, the IC may be underestimated. This issue is explored in the simulation study.

We then define the *estimated instantaneous coupling* between *X* and *Y*, with respect to the base signal *X*, during the interval [*Z*_*i*_, *Z*_*i*+*w*_], where *i* = 1,2,…, *N* − *w*, as the maximum of the cross-correlation between *X* and *Y* over this interval across a range of lags *h*, that is,

(1) $$\begin{array}{cc} & \text{I}{\text{C}}_{X,Y}\left(\left[Z_{i},Z_{i+w}\right]\right)\\ & \;=\underset{h}{\max\;}\frac{\sum_{t=Z_{i}}^{Z_{i+w}}\left(x_{t}-{\bar{x}}_{i}\right)\left(y_{t+h}-{\bar{y}}_{i}\right)}{\sqrt{\sum_{t=Z_{i}}^{Z_{i+w}}{\left(x_{t}-{\bar{x}}_{i}\right)}^{2}\sum_{t=Z_{i}}^{Z_{i+w}}{\left(y_{t+h}-{\bar{y}}_{i}\right)}^{2}}}, \end{array}$$

where

(2) $$\begin{array}{c} {\bar{x}}_{i}=\frac{\sum_{t=Z_{i}}^{Z_{i+w}}x_{t}}{Z_{i+w}-Z_{i}},\;\;{\bar{y}}_{i}=\frac{\sum_{t=Z_{i}}^{Z_{i+w}}y_{t+h}}{Z_{i+w}-Z_{i}}, \end{array}$$

for *i* = 1,2,…, *N* − *w*. In our data analysis, we use the ccf() function in the R package [14] to compute each cross-correlation over the function's default lag range. In general, this range should run a little more than one half-cycle of the base signal in each direction, that is, ≈±(*Z*_*i*+*w*_ − *Z*_*i*_)/*w*, for each window [*Z*_*i*_, *Z*_*i*+*w*_]. This will translate a strong negative correlation into a strong positive one and also ensures that the maximum cross-correlation between the signals will be positive, or very close to zero if negative.

Besides choosing a value for *w*, we also must decide how much overlap between consecutive intervals to allow. Different choices for the overlap parameter will affect the degree of smoothness for the computed IC along the temporal axis, but not its value. We could choose no overlap, so that we compute IC_*X*,*Y*_([*Z*_*i*_, *Z*_*i*+*w*_]) on consecutive adjacent windows [*Z*_1_, *Z*_1+*w*_], [*Z*_1+*w*_, *Z*_1+2*w*_],…. On the other hand, we may choose overlapping windows by selecting some positive integer *m*, with 1 ≤ *m* < *w*, so that IC_*X*,*Y*_([*Z*_*i*_, *Z*_*i*+*w*_]) is computed on overlapping intervals [*Z*_1_, *Z*_1+*w*_], [*Z*_1+*m*_, *Z*_1+*m*+*w*_], [*Z*_1+2*m*_, *Z*_1+2*m*+*w*_],…. In our real data analysis, we select window size *w* = 6 and increment size *m* = 2, so that each consecutive window pair overlaps by two cycles. This choice is empirically made based on considering the apparent duration of synchrony among the plotted signals.

Our method also requires the selection of a base signal. When two gamma signals are synchronized, their time scales are roughly the same, so that they are at about the same frequency. Hence, the IC between *X* and *Y* should be approximately symmetric, so that one will obtain roughly the same IC estimate within each time window, irrespective of which signal is chosen as the base. Any difference will only be due to slightly different measurement windows. The rationale for choosing the base signal depends on whether or not one wishes to analyze the coupling between one specific brain location and several additional locations. If a neuroscientist is only interested in the evolution of synchrony from the perspective of one brain location, then the choice for the base is clear. But, knowing the IC between base signal *X* and signal *Y* and the IC between *X* and a third signal *Y*′ does not provide any information about the IC between *Y* and *Y*′. To determine the latter using our approach, one must choose either *Y* or *Y*′ as the base signal and proceed accordingly. To obtain a complete analysis of the evolution of coupling among all pairs in a set of gamma signals, one would have to repeat the computations with each gamma signal taking its turn as the base. We do not explore this level of analysis in the present paper.

Confidence bounds for the true value of the IC corresponding to each estimated IC measurement may be computed as follows. Let ρ* denote this true value, and let r* denote its estimate. For simplicity let us assume that both values occur at the same lag *h*. If we apply Fisher's *Z*-transformation

(3) $$\begin{array}{c} \zeta =\frac{1}{2}\log\;\left[\frac{1+\rho^{*}}{1-\rho^{*}}\right],\;\;z=\frac{1}{2}\log\;\left[\frac{1+r^{*}}{1-r^{*}}\right], \end{array}$$

and use the established result that the distribution of $\sqrt{n-1}\left(z-\zeta \right)$ approaches that of the standard Gaussian for large values of *n*, we may then compute approximate confidence bounds for *ζ*, and hence for ρ*. That is, an approximate (1 − *α*)100% confidence interval for the true value of the IC is

(4) $$\begin{array}{c} \left(\frac{{\text{e}}^{2L}-1}{{\text{e}}^{2L}+1},\;\;\;\frac{{\text{e}}^{2U}-1}{{\text{e}}^{2U}+1}\right), \end{array}$$

where

(5) $$\begin{array}{c} L=\frac{1}{2}\log\;\left[\frac{1+r^{*}}{1-r^{*}}\right]-\frac{z_{\alpha /2}}{\sqrt{n-1}},\\ U=\frac{1}{2}\log\;\left[\frac{1+r^{*}}{1-r^{*}}\right]+\frac{z_{\alpha /2}}{\sqrt{n-1}}, \end{array}$$

and *z*_*α*/2_ is the 1 − *α*/2 quantile of the standard normal distribution.

Potential problems with this method for obtaining confidence bounds, and an alternative method using extreme value theory that addresses these problems, are discussed in [3].

### 2.2. Identification of Instantaneous Coupling States among Multiple EEG Gamma Signals

We now consider the setting in which we have obtained *J* + 1 EEG gamma signals recorded from tetrodes implanted in different brain regions. Once we have obtained individual estimates of the IC between the selected base signal *X* and each of the *J* signals *Y*_1_,…, *Y*_*J*_ throughout an epoch, we then employ an algorithm to identify neurological states in which particular subsets of these signals are more synchronous with the base signal at any given instant. Essentially, this approach assumes that during each instant different subsets of gamma signals are more synchronized with the base signal than are the remaining signals, based on the interaction among the brain regions in which the electrodes are implanted. The algorithm is used to estimate which subsets of gamma signals are most synchronous with the base signal in each instant, and also to estimate parameters that describe the distribution of the IC estimates among the signals pertaining to each subset. We emphasize that this will provide a model of the synchrony among signals *from the perspective of the selected base signal* only. One may choose additional base signals to obtain models from multiple perspectives and then combine the results. We do not explore the multiple-perspective angle in this paper.

Since the coupling measure we consider here is the maximum cross-correlation between a pair of signals over a range of lags, and hence falls within a bounded interval, we may model the distribution of these maxima with a univariate beta distribution. It is natural to implement a multivariate generalization of the beta distribution to model the joint distribution of the IC among any collection of *J* gamma signals with respect to any base gamma signal. Specifically, we choose the multivariate beta (MVB) distribution, which we derive in the appendix, with parameter vector ***θ*** = (*θ*_1_,…, *θ*_*J*_, *θ*_*J*+1_), *θ*_*j*_ > 0 for *j* = 1,…, *J* + 1. Given the *i*th observation **U**_*i*_ = (*U*_*i*1_,…, *U*_*iJ*_), with 0 < *U*_*ij*_ < 1 for *j* = 1,…, *J*, the joint density of **U**_*i*_ under the MVB distribution is

(6) $$\begin{array}{cc} & f_{U_{i}}\left(u_{i1},\ldots ,u_{iJ}\right)\\ & =\frac{\Gamma \left(\sum_{j=1}^{J+1}\theta_{j}\right)}{\prod_{j=1}^{J+1}\Gamma \left(\theta_{j}\right)}\left(\overset{J}{\underset{j=1}{\prod }}\frac{u_{ij}^{\theta_{j}-1}}{{\left(1-u_{ij}\right)}^{\theta_{j}+1}}\right){\left(1+\overset{J}{\underset{j=1}{\sum }}\frac{u_{ij}}{1-u_{ij}}\right)}^{-\sum_{j=1}^{J+1}\theta_{j}}\;, \end{array}$$

where Γ(*x*) = ∫_0_^*∞*^ *t* ^*x*−1^e^−*t*^d*t*, *x* > 0, is the gamma function.

To implement this model with our IC estimates for *J* gamma signals with respect to a common base signal, we first replace any nonpositive values of the estimated IC with a very small positive value, for example, 0.00001, so that *U*_*j*_ > 0 for each *j*. Likewise, if any estimated IC value equals one, we replace it with 0.99999 so that *U*_*j*_ < 1 for each *j*. In our analysis of real data we obtain very few nonpositive values among the IC estimates, and those that are negative are all very close to zero. To avoid computational errors in the evaluation of (6), due to either large arguments to the gamma function or to computation of the product of very small values, we compute instead the logarithm of the density in (6) then exponentiate the result.

 To estimate the *J* + 1 components of the parameter vector ***θ***, given the *N*′ = *N* − *w* computed IC vectors **U**_*i*_ = (*U*_*i*1_,…, *U*_*iJ*_), *i* = 1,…, *N*′, we maximize the log-likelihood function

(7) $$\begin{array}{cc} & \ell \left(\theta \;|\;U_{1},\ldots ,U_{N^{\prime }}\right)\\ & =N^{\prime }\left[\log\;\Gamma \left(\overset{J+1}{\underset{j=1}{\sum }}\theta_{j}\right)-\overset{J+1}{\underset{j=1}{\sum }}\log\;\Gamma \left(\theta_{j}\right)\right]\\ & \;+\overset{N\prime }{\underset{i=1}{\sum }}\;\overset{J}{\underset{j=1}{\sum }}\left[\left(\theta_{j}-1\right)\log\;U_{ij}-\left(\theta_{j}+1\right)\log\;\left(1-U_{ij}\right)\right]\\ & \;-\left(\overset{J+1}{\underset{j=1}{\sum }}\theta_{j}\right)\overset{N^{\prime }}{\underset{i=1}{\sum }}\log\;\left(1+\overset{J}{\underset{j=1}{\sum }}\frac{U_{ij}}{1-U_{ij}}\right) \end{array}$$

over all ***θ*** ∈ (0, *∞*)^*J*+1^. This computation is carried out using the Expectation Maximization (EM) algorithm [12].

Next, we postulate that the *N*′ IC estimates may be grouped into distinct IC states. By *IC states* we mean occasions in which specific subsets of the *J* gamma signals have an IC with the base signal which is relatively high, perhaps above some threshold. If we think of each signal as being either coupled or not coupled with the base signal during any instant, based on some threshold, there would be 2^*J*^ possible IC states. However, we expect that tetrodes located near each other should tend to exhibit relatively equivalent IC levels with respect to any base, so that far fewer distinct states actually occur.

We assume there are *p* ≥ 1 such IC states among the IC estimates **U**_1_,…, **U**_*N*′_, and then use a clustering procedure to estimate parameters that describe the distinct states and to classify the individual **U**_*i*_ among them. One possible clustering procedure is the *k*-means algorithm, in which the vectors **U**_1_,…, **U**_*N*′_ are randomly assigned among *p*  clusters, and the mean vector for each cluster is computed. The algorithm then reassigns each vector, if necessary, to the cluster whose mean is nearest, in terms of some distance measure. Since the clusters are usually altered by the reassignments, the algorithm recomputes the mean vector of each cluster and then conducts any necessary reassignments of vectors to nearer cluster means. This process continues iteratively until no reassignments are necessary (or a maximum number of iterations is reached). The R version of the *k*-means algorithm implements by default the method described in [15]. This method ensures that *p* clusters are returned. Because the algorithm is sensitive to the initial allocation, it should be run multiple times, and the best result should be chosen in terms of minimum error sum of squares. However, we choose a more sophisticated approach that takes into account the apparent MVB distribution of the IC estimates.

Since we assume the representative IC vectors **U**_1_,…, **U**_*N*′_ follow an MVB distribution, we implement a mixture model which assumes that each **U**_*n*_ arises independently from one of *p* IC states, each of which follows its own MVB distribution with its own parameter vector ***θ***_*k*_ = (*θ*_*k*,1_; *θ*_*k*,2_; …; *θ*_*k*,*J*+1_) for *k* = 1,…, *p*. This model uses the MVB density to assign each **U**_*i*_ to that IC state for which the probability that belongs to that state is largest. These probabilities are latent parameters which must be estimated along with the parameter vectors ***θ***_1_,…, ***θ***_*p*_ corresponding to the respective IC states. The EM algorithm [12] is suited to this purpose. The independence assumption is approximate here, but we rely on the robustness of our estimation procedure when the sample size *N*′ is quite large.

To implement the EM algorithm, we let *P*_***θ***_*k*__(**u**_*i*_) denote the value of the MVB density corresponding to the *k*th state evaluated at IC estimate **u**_*i*_ = (*u*_*i*1_,…, *u*_*iJ*_), where *i* = 1,…, *N*′, and we let *π*_*k*_ denote the probability that a randomly selected IC estimate belongs to IC state *k*. In the E-step of the EM algorithm, we determine the *responsibility* of the *k*th state for **U**_*i*_ at the *q*th iteration, *q* = 1,2,…, by computing

(8) $$\begin{array}{c} r_{k,i}^{\left(q\right)}=\frac{\pi_{k}^{\left(q-1\right)}P_{\theta_{k}^{\left(q-1\right)}}\left(u_{i}\right)}{\sum_{l=1}^{p}\pi_{l}^{\left(q-1\right)}P_{\theta_{l}^{\left(q-1\right)}}\left(u_{i}\right)} \end{array}$$

for *i* = 1,…, *N*′ and *k* = 1,…, *p*. Then in the M-step, we estimate the mixing parameters and the distribution parameters at the *q*th iteration by computing

(9) $$\begin{array}{c} \pi_{k}^{\left(q\right)}=\frac{1}{N^{\prime }}\overset{N\prime }{\underset{i=1}{\sum }}r_{k,i}^{\left(q\right)},\\ \theta_{k}^{\left(q\right)}=\underset{\theta \in \Theta }{\text{argmax}}\overset{N^{\prime }}{\underset{i=1}{\sum }}r_{k,i}^{\left(q\right)}\log\;P_{\theta }\left(u_{i}\right), \end{array}$$

for *k* = 1,…, *p*. We then continue to iterate between the E-step and the M-step until the parameter estimates converge within a prespecified tolerance. In our real data analysis, we use the constrOptim() procedure in R with appropriate settings in order to find the optimal value of ***θ*** in the M-step.

The success of the EM algorithm is sensitive to the selection of the initial estimates *π*_*k*_^(0)^ and ***θ***_*k*_^(0)^. In our data analysis, we use the *k*-means algorithm to obtain *p* clusters of our IC estimates, then find maximum likelihood estimates (MLEs) of the univariate beta parameters for each individual dimension of the *J*-dimensional IC vector for each of the *p* clusters. We combine these univariate estimates into one parameter estimate ***θ***_*k*_ for each cluster, and use the constrOptim() procedure to determine the MLE of ***θ***_*k*_ for each cluster. Then, *π*_*k*_^(0)^ is the proportion of IC estimates assigned to the *k*th cluster, and ***θ***_*k*_^(0)^ is the computed MLE, for *k* = 1,…, *p*. In general, we find that this approach consistently provides us with initial estimates that lead to eventual convergence to an optimal solution.

In order to implement the above procedure, it is necessary to determine the number *p* of IC states that occur among the gamma signals. We make use of the Bayes Information Criterion (BIC) to estimate the true number of clusters *p* that are represented in the data, if in fact the data are clustered. That is, we perform the EM algorithm for *p* = 2,3,…, and for each value of *p*, we obtain optimal parameter estimates *π*_k_* and ***θ***_k_* for *k* = 1,…, *p* and compute the *mixture log-likelihood* (see [16]) at these optimal parameter estimates as follows:

(10) $$\begin{array}{cc} \ell_{\text{mix}} & =\ell \left(\theta_{1}^{*},\ldots ,\theta_{p}^{*},\pi_{1}^{*},\ldots ,\pi_{p}^{*}\;|\;u_{1},\ldots ,u_{N^{\prime }}\right)\\ & =\overset{N^{\prime }}{\underset{i=1}{\sum }}\log\;\left[\overset{p}{\underset{k=1}{\sum }}\pi_{k}^{*}P_{\theta_{k}^{*}}\left(u_{i}\right)\;\right]. \end{array}$$

The BIC for the model is then computed from

(11) $$\begin{array}{c} \text{BI}{\text{C}}_{p}=-2\ell_{\text{mix}}+\left[p\left(J+2\right)-1\right]\log\;\left(N^{\prime }\right), \end{array}$$

where *J* is the number of gamma signals. (Note that for each of the *p* states we estimate a (*J* + 1)-dimensional parameter vector ***θ***_*k*_ and a mixing probability *π*_*k*_, for a total of *p*(*J* + 2) estimated parameters. However, once *π*_1_,…, *π*_*p*−1_ are estimated, the value of *π*_*p*_ is then fixed, since the mixing probabilities sum to one, resulting in *p*(*J* + 2) − 1 independent estimated parameters). We would thus choose that mixture model, over a suitable range of values for *p*, for which the value of the BIC is minimized. That is, we conclude that the IC with the base signal among the other *J* signals varies among P* distinct states, where

(12) $$\begin{array}{c} p^{*}=\underset{p}{\text{argmin}}\;\text{BI}{\text{C}}_{p}. \end{array}$$

We may then form an IC state sequence corresponding to the zero crossings of the base signal which estimates which sets of EEG gamma signals are synchronized with the base signal within each measurement window as time unfolds.

To summarize, our procedure is as follows.

1. Choose one signal as the base among *J* + 1 gamma signals recorded at tetrodes implanted in different brain regions.
2. Compute the estimated IC between the base signal and the other *J* signals within a sliding variable-length window throughout an epoch of activity. This results in a time-ordered sequence of *J*-dimensional IC estimates.
3. For any block of the sequence, use the EM algorithm to maximize the log-likelihood based on a mixed multivariate beta model involving *p* distinct clusters, where *p* ranges over a set of values.
4. For each value of *p*, use the optimized log-likelihood to assign each IC estimate to one of *p* IC states and to obtain estimates for the model parameter vector corresponding to each IC state.
5. Select the model for which the BIC is minimized. This model yields an IC state sequence which represents the evolution in the coupling of the different brain regions among the *p* IC states from moment to moment from the perspective of the selected base signal.
6. Since the sequence of IC estimates, and the subsequent IC state sequence, correspond to the zero crossings of the base signal, we may map the IC states back to the time scale of the EEG gamma signals.

## 3. Results

### 3.1. Simulation Studies

**Figure 1:**
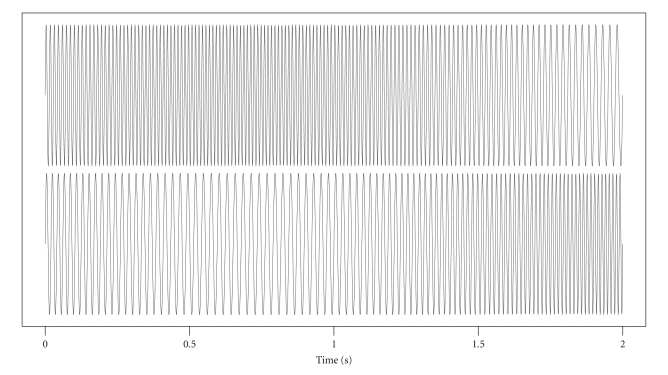
Simulated EEG gamma signals *X*(*t*) (top) and *Y*(*t*) (first two seconds).

To demonstrate the benefits of computing estimates of the instantaneous coupling between two EEG gamma signals using a variable window, we generate two signals

(13) $$\begin{array}{c} X\left(t\right)=\sin\left\{2\pi \left[70+10\sin\left(0.5\pi t\right)\right]t\right\},\\ Y\left(t\right)=\sin\left\{2\pi \left[50+10\sin\left(0.5\pi \left(t-2\right)\right)\right]t\right\}, \end{array}$$

where *t* varies from 0 to 20 seconds at a resolution of 1500 points per second. This resolution mimics that of the real EEG data. A plot of the first two seconds of this signal pair is shown in Figure 1. We intend to show that, using a variable window, with the parameter *w* well-tuned, the IC computation is close to one when the two signals are at about the same frequency, plus or minus a small lag, and close to zero otherwise. 

Signal *X* has an instantaneous frequency of 70 + 10sin(0.5*π* *t*) that oscillates between 60 Hz and 80 Hz, while the instantaneous frequency 50 + 10sin(0.5*π*(*t* − 2)) of signal *Y* oscillates between 40 Hz and 60 Hz. Both signals achieve the instantaneous frequency of 60 Hz simultaneously. However, the *observed* frequency of each signal within any time interval—that is, the number of cycles the signal actually exhibits per discrete time unit—is *not* the same as its instantaneous frequency when the instantaneous frequency is nonconstant, as here. To compute an estimate of the observed frequency of either signals at each time *t*, we count the number of zero crossings in the interval [*t* − 0.05, *t* + 0.05]. Since every two zero crossings represents one cycle, we divide this count by two. Dividing by 0.1 then gives us an estimate of the observed frequency of the signal at time *t*. For our purposes, we do not need this value for the first or last 0.05 seconds of the 20-second epoch. The observed frequency for signal *X* thus ranges between 0 Hz and 375 Hz, with a median of 95 Hz, while the observed frequency for *Y* ranges between 0 Hz and 340 Hz, with a median of 85 Hz. While these frequency ranges exceed the gamma band, the benefits of the IC computation method that we intend to demonstrate in this simulation study do not compel us to focus on any particular frequency band.

**Figure 2:**
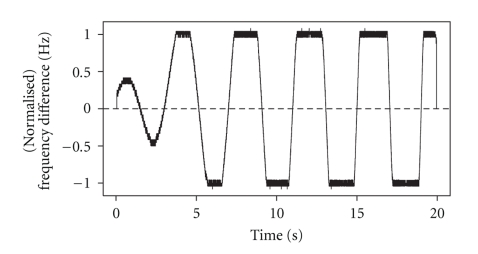
Scaled difference in average frequency between *X* and *Y* throughout the epoch.

We subtract the observed frequency of *Y* from that of *X*, resulting in a difference which ranges in absolute value between 0 Hz and 130 Hz, with a median of 120 Hz. Then, so that we will be able to plot the computed IC alongside the frequency difference and to make comparisons, we divide the difference in observed frequencies by 130 in order to place the difference on a scale of −1 to 1. A plot of this normalized frequency difference is displayed in Figure 2. The computed IC between *X* and *Y* should be equal to one at points where this plot crosses the horizontal axis, as it is around these points (plus or minus a small lag) that the two simulated gamma signals become synchronized. The IC should fall to zero elsewhere. We note that the duration of the synchronization in this simulation does not necessarily last for several cycles, as is assumed for the EEG gamma signals. Again, the benefits that we intend to demonstrate in this simulation study will be carried over to the EEG data analysis without requiring us to mimic the lengthier duration of synchrony. 

**Figure 3:**
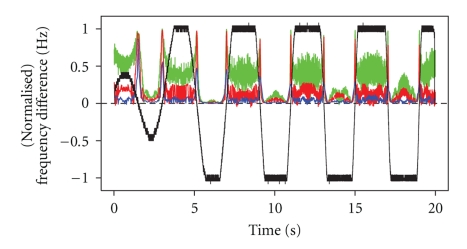
Estimated IC when *w* = 3 (green), *w* = 6 (red), and *w* = 18 (blue), with the scaled difference in average frequency between *X* and *Y* throughout the epoch.

We apply the variable window technique described in Section 2.1 to estimate the IC between signals *X* and *Y*, with *X* chosen as the base signal, using three different values for *w*, where *w* is the number of half cycles of the base signal, to determine each measurement window. In each case, we choose *m* = *w*/3 as the increment size. The results are mapped back to the time scale of the signals, and displayed in Figure 3 along with the normalized frequency difference from Figure 2. With *w* = 3, the estimated IC is close to one whenever the frequency difference is near zero, as desired, but it oscillates around 0.5 in several intervals where it should be near zero (Figure 3, green curve). When *w* = 18, the estimated IC is near zero when the signals are asynchronous, but not close to one when they are synchronized (Figure 3, blue curve). When *w* = 6, the result is most promising, since the estimated IC is close to zero whenever the frequency difference is large, and close to one when the two signals are synchronized (Figure 3, red curve). 

**Figure 4:**
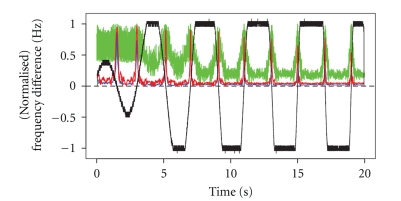
Estimated IC with fixed windows of 18 (green), 90 (red), and 210 (blue) time points, with the scaled difference in average frequency between *X* and *Y* throughout the epoch.

For comparison, we estimate the IC between *X* and *Y* in the same manner, but using windows of fixed width (Figure 4). When the window size is 18 time points, the estimated IC is close to one when the signals are synchronized, but is not anywhere near zero in the first few intervals in which the signals are asynchronous (Figure 4, green curve). Also, when it does approach zero, it does not drop sharply, but falls off gradually. When the window size is 210 time points, the estimated IC is near zero when it should be, but it is generally much too small when it should be close to one (Figure 4, blue curve). When the window size is 90 time points, we obtain the best IC estimate of the three (Figure 4, red curve). But, we note that at several time points where it should be close to one it is closer to 0.5. 

This simulation demonstrates that whether a variable window or a fixed window is used, estimation of the IC between two signals is highly sensitive to the choice of the parameter that affects the window width. But the variable window approach has the advantage of adaptability to the local frequency, so that once a good choice is made for the tuning parameter, the IC estimate will be consistently reliable as the signal frequency varies throughout an epoch. A choice for the fixed window width may work well for a specific frequency range, but will not perform well outside that range. In the simulated signals, the range for the average effective frequency of the base signal *X* grows as the time increases from 0 to 20 seconds. Hence, the performance of the IC estimate when *w* = 18 improves as time passes (Figure 4, green), while the performance of the IC estimates when *w* = 90 and *w* = 210 diminishes (Figure 4, red and blue, resp.). With the variable-window approach (Figure 3), we see that the performance of each IC estimate remains consistent throughout the epoch even though the range of the average frequency changes. Hence, once we identify a good choice for *w*, we can employ it in the study of any oscillating signal. Based on our analysis, it seems that *w* = 6 is a good choice.

### 3.2. Experimental Data: EEG Gamma Signals from the Rat Hippocampus

To demonstrate our method, we analyze EEG data recorded from nine tetrodes located in the hippocampal formation of rats before, during and after they perform an exercise on a track. Tetrodes were placed in the medial entorhinal cortex (MEC; four electrodes) and CA1 region (five electrodes). For our analysis, we focus on the EEG during a “rest” epoch, when the rat is in its cage (but not necessarily inactive) after one exercise epoch and before the next.

**Figure 5:**
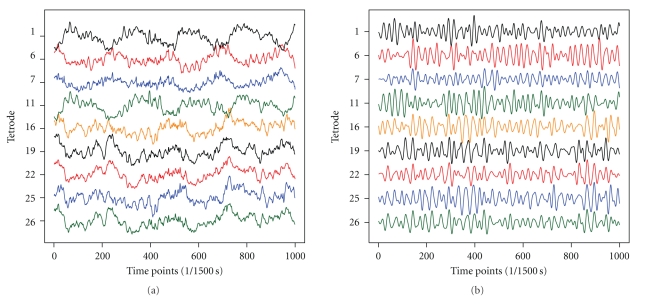
Raw EEG signals and filtered gamma signals from MEC (1,6,7,11) and CA1 (16,19,22,25,26) of rat during the first 2/3 second of a rest epoch.

A typical data set consists of approximately 15 to 20 minutes of EEG data recorded at 1.5 kHz. Our investigation focuses on the identification of instants when the gamma rhythms in both regions become synchronized on a short time scale. As an initial preprocessing step, the raw EEG signals were filtered in the 40–100 Hz range using the filtfilt.m routine in Matlab. This process was used to extract the gamma signal from the raw EEG signal. Figure 5 shows nine raw and nine filtered signals, respectively, from the first 2/3 second of a recording epoch. Note that the cycles for the filtered signals do not always cross the horizontal axis. Hence, for any gamma signal that we might select for a base signal, its cycles are not necessarily identifiable by zero crossings. However, the Hilbert transform is applied to each filtered signal to obtain the Hilbert phase at each time point, so that we may identify the cycles by locating the points where the phase is approximately ±*π*/2.

**Figure 6:**
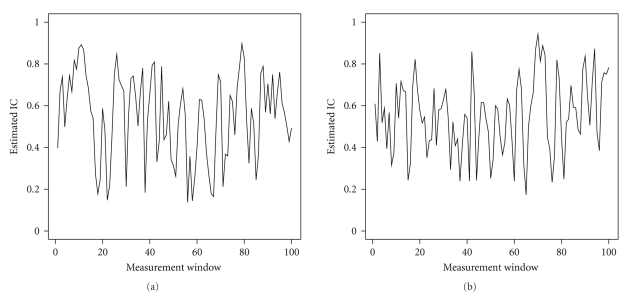
First 100 instantaneous coupling estimates between gamma signals at Tetrodes 1 (base signal) and 6 (a), and Tetrodes 1 (base signal) and 26 (b), at the beginning of an epoch.

For this analysis, we select the gamma signal extracted from Tetrode 1, located in the MEC, as the base signal. Figure 6 displays plots of the first 100 estimated IC values between this base gamma signal and the gamma signals at Tetrodes 6 and 26, respectively, at the beginning of a recording epoch, using *w* = 6 and *m* = 2. Note that the horizontal axis is not transformed back to the time scale, but is given in terms of the sequence of measurement windows. We observe that the estimated IC alternates between values above 0.6 and values below 0.4 in each plot. Frequent instants of high synchrony are expected in the left plot since Tetrodes 1 and 6 are both located within the MEC, albeit in different parts, while instants of high synchrony should be less frequent in the right plot, since Tetrode 26 is not in the MEC. For any given time span we may choose any of the nine available gamma signals as the base signal and use the remaining signals to compute an eight-dimensional time series of IC estimates corresponding to that base signal, using any appropriate values of *w* or *m*. In this section, we explore whether the time series can be partitioned into specific IC states.

**Figure 7:**
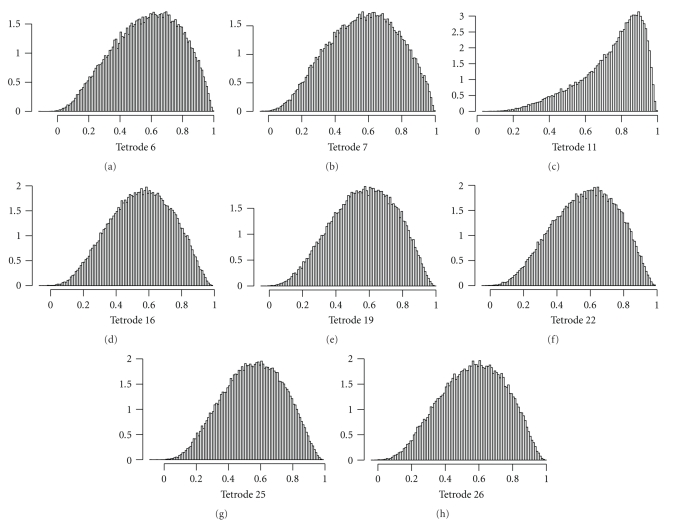
Distributions of estimated instantaneous coupling between gamma rhythms at Tetrode 1 and each of the other eight tetrodes during a rest epoch.

Figure 7 shows eight histograms of the distributions of the 74,490 estimated IC values between Tetrode 1 and each of the other eight tetrodes during a twenty-minute rest epoch. Almost all of the estimated IC values are distributed between zero and one, with a negligible remainder just slightly below zero. Hence, the MVB distribution should be appropriate for modeling these data, as discussed in Section 2.2. 

**Figure 8:**
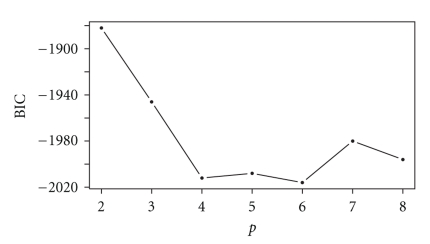
Progression of BIC as *p* increases, for EEG application.

Our goal is to reduce this eight-dimensional time series to a single dimension by identifying distinct IC states when different subsets of signals are coupled with the base. This single dimension—the IC state—will take one of the values in {1,…, p*} in each measurement window, where p* is defined in (12). The histograms in Figure 7 may be considered superpositions of the histograms of the IC estimates corresponding to these distinct states, each of which is modeled by an MVB distribution. We implement the EM algorithm as described in Section 2.2 to determine the parameters of these distributions and to determine the state membership of each IC estimate, using the R statistical package. To illustrate our method, we consider only a ten-second block from the full recorded time series, corresponding to 746 consecutive and overlapping three-cycle windows of the base signal. Moreover, we work with only four dimensions rather than all eight, by choosing representatives from each location in the brain in which multiple EEG signals are recorded. Using the IC estimates between the base signal at Tetrode 1 and the signals at Tetrodes 6, 11, 25, and 26, computed during an interval when the rat is in its cage, we proceed with the EM algorithm. In Figure 8 we note that the BIC decreases as *p* increments from 2 to 4, is relatively constant for *p* in the range of 4 to 6 states, and then increases thereafter. We conclude that the instantaneous coupling between the base signal recorded in the MEC and the signals from the four other selected locations transition among four to six distinct IC states during the chosen ten-second block. Since the simplest model is preferred, we adopt a model consisting of four IC states.

We run the EM algorithm for values of *p* in the range 2 ≤ *p* ≤ 8. Once the algorithm converges, we use the parameter estimates to compute the mixture log-likelihood according to (10) and then obtain the BIC according to (11). We implement the algorithm simultaneously for the seven values of *p*, using several 3.06 GHz machines. Model estimation required from one to eleven days as *p* increased in value. The parameter estimates corresponding to each of these four IC states, resulting from 108 iterations of the EM algorithm are shown in Table 1.

 To assign each IC estimate to its appropriate state, we inspect the corresponding vector of responsibilities returned by the EM algorithm after its convergence (see (8) with *p* = 4) and note the position of the largest value, that is, we assign the *i*th IC estimate to state *k*, where

(14) $$\begin{array}{c} k=\underset{j}{\text{argmax}}\left\{r_{j,i}\;|\;j=1,2,3,4\right\}, \end{array}$$

for *i* = 1,…, 746. We then obtain the mean vector of the subsets of IC estimates assigned to each of the four states, along with their corresponding standard deviations as shown in Table 2.

**Figure 9:**
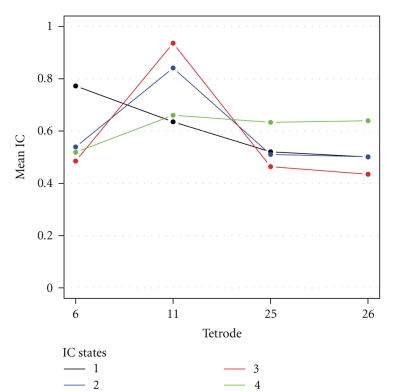
Means of the subsets of IC estimates corresponding to each of the four IC states, with the base signal at Tetrode 1, based on optimized mixed MVB model.

 We plot the group means in Figure 9. 

If we use 0.6 as the threshold for distinguishing coupling from noncoupling, then State 1, which comprises 16.2% of the block of IC estimates, represents instants when the base signal is coupled with the other two MEC signals, but not with the CA1 signals. State 4, comprising 37.6% of the IC estimates, represents occasions when the base signal is coupled with the signal at nearby Tetrode 11 in the MEC and with the signals at Tetrodes 25 and 26 in the CA1, but not with the signal at Tetrode 6 in another part of the MEC. Hence, State 4 indicates synchronization between the MEC and the CA1 cell layer in the rat's brain. States 2 and 3, comprising 39.6% and 6.6% of the IC estimates, respectively, represent instants when the base signal is only coupled with the gamma signal at neighboring Tetrode 11, although at different levels. Since the mean vectors for the subsets of IC estimates corresponding to these two states appear to be very similar, and less than 7% of the estimates are assigned to State 3, we may consider combining them. However, what distinguishes the two states is not the mean vectors of the corresponding subsets of IC estimates, but the parameter estimates for the two states. The estimates of the parameter *θ*_2_ for States 2 and 3 are very different, which implies that the IC estimates for these two states are distributed quite differently. Moreover, the BIC criterion clearly guides us to a minimum of four distinct IC states.

**Figure 10:**
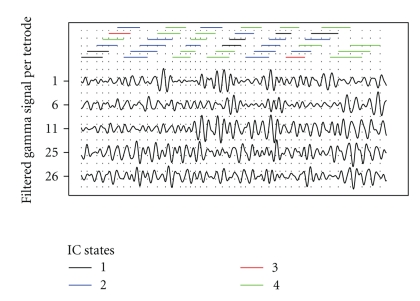
Gamma signals during a 2/3-second block of a recording epoch, with IC state designation indicated for each window when the base signal is at Tetrode 1 and four IC states are modeled. Vertical dotted lines correspond to computed cycles of the base signal.

Since we have performed this analysis only on one block of IC estimates taken from a rest epoch, we cannot assume that our results apply to the entire epoch or to any other time period. We may repeat the estimation using a block of similar size from an interval during which the rat is motionless in its cage, and may be asleep, or when the rat is performing tasks on the track. Moreover, these procedures may be applied when the EEG signal at any tetrode is selected as the base signal, so that occasions of coupling between any two brain regions can be identified. This approach thus enables a neuroscientist to identify instants when different brain regions are synchronized, and investigate the correspondence between the frequency of such instants and behavioral covariates. Figure 10 shows the gamma signals extracted from the EEG recordings at Tetrodes 1, 6, 11, 25, and 26 at the beginning of the sample block on which our method was applied, along with a colored bar above each window associated with three cycles of the base signal at Tetrode 1. The colored bars indicate to which of the four IC states the signals are assigned by our method at each instant. The colors and their corresponding states match those designated in Figure 9, that is, black bars indicate coupling between the Tetrode 1 signal and the signals at Tetrodes 6 and 11 in the MEC; blue bars and red bars denote coupling between the Tetrode 1 signal and the nearby signal at Tetrode 11; green bars denote coupling between the Tetrode 1 signal and the signals at Tetrodes 11, 25, and 26. Both the sequence and frequency of IC states within any interval may be meaningful in future investigations. 

## 4. Discussion

We have developed a computational method for estimating the short time-scale coupling between gamma signals filtered from two EEG recordings, along with confidence bounds on the estimate. This computation requires the selection of one signal as the base and partitioning the recording epoch based on the cycles of that base. We define the instantaneous coupling (IC) on any measurement window as the maximum over all lags of the cross-correlation between the base and the other signal during that window. This value is computed throughout the epoch on a sliding window consisting of three cycles of the base, incrementing one cycle at a time so that consecutive measurements come from overlapping windows. At instants when the coupling between signals is strong, our IC estimate should be in the 0.6 to 1.0 range. A simulation study confirms that this procedure is sufficiently accurate in identifying instants of low and high synchronization.

When we choose one gamma signal as the base and compute the IC between that signal and the other gamma signals at each of the other tetrodes, we obtain a distribution of IC estimates which may be jointly modeled using the multivariate beta (MVB) distribution. The parameters of this distribution may be estimated using the EM algorithm. We can examine the structure of the set of IC estimates by implementing a mixture model with a preselected number of clusters representing IC states among the estimates. If the estimates naturally fall into a particular number of clusters, the Bayes Information Criterion will guide us to that number. This enables us to identify instants in which subsets of the gamma signals are synchronized with the chosen base signal.

The method we outline allows neuroscientists to detect the evolution of IC on a short time scale among multi-dimensional EEG gamma waves. This evolution reveals the trajectory of local synchronous patterns and could be used to identify binding between separate parts of the brain. These evolving patterns of local and global synchrony may provide a platform for scientists to map out moment-by-moment progression of signal transmission pathways among distinct regions of the brain. Our methodology addresses several algorithmic challenges. First, the chosen measurement window of three cycles of a base gamma signal seems to reasonably capture such instantaneous synchronization, which typically lasts for fewer than five cycles. We believe that this is a natural timescale for such synchronization manifested through gamma rhythms. Secondly, the mixed MVB model with parameters estimated by the EM algorithm provides an effective and reliable tool for identifying IC states that signify the occurrence of synchronization among different regions of the brain.

One outstanding issue is the computational burden of the EM algorithm, which is known to converge slowly. In our EEG example we limited our analysis to a ten-second block and only four of the eight dimensions, and it took eleven days to converge. However, the computational efficiency would be greatly improved by using custom code, so that the method could be applied effectively to more dimensions and a much longer time span. Alternate methods for model fitting, including Markov chain Monte Carlo techniques or the simulated annealing algorithm, may also be developed. Our focus in this paper is the illustration of our method, rather than optimizing the computational efficiency. This task will be undertaken in future work.

## Figures and Tables

**Table 1:** 

State	*π*	*θ*_1_	*θ*_2_	*θ*_3_	*θ*_4_	*θ*_5_
1	0.16	8.4	4.7	3.1	2.9	2.9
2	0.40	3.2	13.6	2.8	2.8	2.7
3	0.07	2.8	39.2	2.6	2.2	2.8
4	0.38	2.1	3.8	3.2	3.2	1.9

**Table 2:** 

State	Mean (standard deviation)			
	Tetrode 6	Tetrode 11	Tetrode 25	Tetrode 26
1	0.77	0.64	0.52	0.50
	(0.08)	(0.16)	(0.16)	(0.15)

2	0.54	0.84	0.51	0.50
	(0.17)	(0.07)	(0.17)	(0.16)

3	0.48	0.94	0.46	0.43
	(0.17)	(0.03)	(0.16)	(0.19)

4	0.52	0.66	0.63	0.64
	(0.17)	(0.16)	(0.17)	(0.17)

## Appendix

Our derivation of the multivariate beta (MVB) density follows the approach of Olkin and Liu [17]. We include here a full derivation due to typographical errors found in the formula for the multivariate case presented in their original publication. Let *X*_1_ ~ Γ(*θ*_1_, 1),…, *X*_*J*_ ~ Γ(*θ*_*J*_, 1) and *Y* ~ Γ(*θ*_*J*+1_, 1) be independent gamma random variables, where Γ(*θ*_*j*_, 1) denotes the standard gamma distribution with shape parameter *θ*_*j*_. Hence the joint density of *X*_1_,…, *X*_*J*_, *Y* is

(A.1) $$f\left(x_{1},\ldots ,x_{j},y\right)=\frac{\prod_{j=1}^{J}x_{j}^{\theta_{j}-1}y^{\theta_{J+1}-1}\exp\;\left\{-\left(\sum_{j=1}^{J}x_{j}+y\right)\right\}}{\prod_{j=1}^{J+1}\Gamma \left(\theta_{j}\right)},$$

where Γ(·) denotes the gamma function and *x*_1_,…, *x*_*J*_, *y* are all positive. Define

(A.2) $$U_{1}=\frac{X_{1}}{X_{1}+Y},\ldots ,U_{J}=\frac{X_{J}}{X_{J}+Y},$$

so that 0 < *U*_*j*_ < 1 for *j* = 1,…, *J*, and correlation among *U*_1_,…, *U*_*j*_ is established by the common dependence on *Y*. Through simple algebra, we have

(A.3) $$X_{j}=\frac{YU_{j}}{1-U_{j}}\;\text{for}\;\;j=1,\ldots ,J.$$

Hence

(A.4) $$\begin{array}{c} \frac{\partial x_{j}}{\partial u_{j}}=\frac{y}{{\left(1-u_{j}\right)}^{2}},\;\frac{\partial x_{j}}{\partial y}=\frac{u_{j}}{1-u_{j}}\;\text{for}\;\;j=1,\ldots ,J,\\ \frac{\partial x_{j}}{\partial u_{k}}=0\;\text{for}\;\;j\neq k, \end{array}$$

while

(A.5) $$\frac{\partial y}{\partial u_{j}}=0\;\text{for}\;\;j=1,\ldots J,\;\;\frac{\partial y}{\partial y}=1,$$

giving us the Jacobian matrix

(A.6) $$\begin{array}{c} \left[\begin{array}{ccccc} \frac{y}{{\left(1-u_{1}\right)}^{2}} & 0 & \cdots & 0 & \frac{u_{1}}{1-u_{1}}\\ 0 & \frac{y}{{\left(1-u_{2}\right)}^{2}} & \cdots & 0 & \frac{u_{2}}{1-u_{2}}\\ \vdots & \vdots & \ddots & \vdots & \vdots \\ 0 & 0 & \cdots & \frac{y}{{\left(1-u_{J}\right)}^{2}} & \frac{u_{J}}{1-u_{J}}\\ 0 & 0 & \cdots & 0 & 1 \end{array}\right], \end{array}$$

whose determinant is just the product of the diagonal entries,

(A.7) $$\frac{y^{J}}{\prod_{j=1}^{J}{\left(1-u_{j}\right)}^{2}}.$$

Therefore the joint density of *U*_1_,…, *U*_*J*_, *Y* is

(A.8) $$\begin{array}{cc} f\left(u_{1},\ldots ,u_{J},y\right) & =\frac{y^{J}}{\prod_{j=1}^{J}{\left(1-u_{j}\right)}^{2}}\overset{J}{\underset{j=1}{\prod }}\frac{{\left(yu_{j}\right)}^{\theta_{j}-1}}{{\left(1-u_{j}\right)}^{\theta_{j}-1}}y^{\theta_{J+1}-1}\\ & \;\times \frac{\exp\;\left\{-\left(\sum_{j=1}^{J}\left(yu_{j}/\left(1-u_{j}\right)\right)+y\right)\right\}}{\prod_{j=1}^{J+1}\Gamma \left(\theta_{j}\right)}\\ & =\frac{\prod_{j=1}^{J}u_{j}^{\theta_{j}-1}y^{\sum_{j=1}^{J+1}\theta_{j}-1}}{\prod_{j=1}^{J}{\left(1-u_{j}\right)}^{\theta_{j}+1}\prod_{j=1}^{J+1}\Gamma \left(\theta_{j}\right)}\\ & \;\times \exp\;\left\{-y\left(1+\overset{J}{\underset{j=1}{\sum }}\frac{u_{j}}{1-u_{j}}\right)\right\}. \end{array}$$

Let

(A.9) $$q=1+\overset{J}{\underset{j=1}{\sum }}\frac{u_{j}}{1-u_{j}},\;\;\theta =\overset{J+1}{\underset{j=1}{\sum }}\theta_{j}.$$

We obtain the joint density of *U*_1_,…, *U*_*J*_, which is our objective, by integrating out *y* as follows:

(A.10) $$\begin{array}{cc} & f\left(u_{1},\ldots ,u_{J}\right)\\ & =\frac{\prod_{j=1}^{J}u_{j}^{\theta_{j}-1}}{\prod_{j=1}^{J}{\left(1-u_{j}\right)}^{\theta_{j}+1}\prod_{j=1}^{J+1}\Gamma \left(\theta_{j}\right)}\overset{\infty }{\underset{0}{\int }}y^{\theta -1}{\text{e}}^{-qy}\text{d}y\\ & =\frac{\Gamma \left(\theta \right)}{\prod_{j=1}^{J+1}\Gamma \left(\theta_{j}\right)}\frac{1}{q^{\theta }}\overset{J}{\underset{j=1}{\prod }}\frac{u_{j}^{\theta_{j}-1}}{{\left(1-u_{j}\right)}^{\theta_{j}+1}}\\ & =\frac{\Gamma \left(\sum_{j=1}^{J+1}\theta_{j}\right)}{\prod_{j=1}^{J+1}\Gamma \left(\theta_{j}\right)}\left(\overset{J}{\underset{j=1}{\prod }}\frac{u_{j}^{\theta_{j}-1}}{{\left(1-u_{j}\right)}^{\theta_{j}+1}}\right){\left(1+\overset{J}{\underset{j=1}{\sum }}\frac{u_{j}}{1-u_{j}}\right)}^{-\sum_{j=1}^{J+1}\theta_{j}}, \end{array}$$

where 0 < *u*_*j*_ < 1 for *j* = 1,…, *J*. This is the multivariate beta density given in (6).
